# Utility of Serum Anti-Müllerian Hormone Measurement as Part of Polycystic Ovary Syndrome Diagnosis

**DOI:** 10.1055/s-0044-1786731

**Published:** 2024-05-22

**Authors:** Terhi T. Piltonen, Johanna Viita-aho, Ulla Saarela, Johanna Melin, Maria Forslund

**Affiliations:** 1 Department of Obstetrics and Gynecology, Research Unit of Clinical Medicine, Medical Research Center Oulu, University of Oulu, Oulu University Hospital, Oulu, Finland; 2 Department of Obstetrics and Gynecology, University of Helsinki, Helsinki University Hospital, Helsinki, Finland; 3 Department of Obstetrics and Gynecology, Institute of Clinical Sciences, Sahlgrenska Academy, University of Gothenburg, Gothenburg, Sweden

**Keywords:** PCOS, PCOS diagnosis, AMH

## Abstract

The 2023 international evidence-based guideline update for the assessment and management of polycystic ovary syndrome (PCOS) recommends using the Rotterdam criteria for the diagnosis of PCOS. The updated guideline has evidence-based recommendation for the diagnosis, and it now also includes serum anti-Müllerian hormone (AMH) measurement as an alternative tool for gynecological ultrasound to diagnose polycystic ovary morphology (PCOM). The aim of this new recommendation was to facilitate PCOS diagnostic workup in primary care and other disciplines, as currently most diagnosing is done in gynecology and infertility clinics. Here, we review factors affecting AMH levels as well as the utility of AMH in PCOS diagnosis. We identified relevant studies that report different cut-offs for AMH to diagnose PCOM as part of PCOS diagnosis. There are, however, some limitations when using AMH that should be acknowledged. These include physiological aspects like age, ethnicity, and obesity and iatrogenic causes like hormonal medication and ovarian surgery. Also reference ranges are different depending on AMH assay used. As a summary, we conclude that AMH is a usable tool in PCOM diagnostics, but it does not have a single cut-off. Therefore, further studies are needed to establish age and assay-based reference ranges.

## Polycystic Ovary Syndrome Presents with Vast Morbidity

Polycystic ovary syndrome (PCOS) is the most common endocrinopathy in women, affecting one woman out of eight worldwide. 1 2 The manifestation of PCOS is a result of the cumulative impact of altered genetic, epigenetic, and protein profiles, leading to systemic dysfunction. 3 PCOS is associated with metabolic, reproductive, and psychological features, and women with PCOS have an increased prevalence of conditions such as subfertility, metabolic syndrome, and cardiovascular diseases. 3 Moreover, depressive and anxiety symptoms are four to five times more prevalent and long lasting in women with PCOS, accompanied by additional psychological characteristics, including disordered eating and body image, conditions that all cause significant reduction in quality of life. 4 5 PCOS also increases pregnancy-related risks, including miscarriages, gestational diabetes, pregnancy-induced hypertension, preeclampsia, and premature delivery. 1 6 Given the high risk for several comorbidities, women with PCOS and young individuals with or at risk of PCOS should be identified from the population early on to enable early prevention and support, including during pregnancy. Moreover, by educating healthcare professionals and patients, some of the medical, psychosocial, and economic burdens associated with PCOS could be prevented including its related comorbidities. 7 However, there is a delicate balance between under- and overdiagnosis. Underdiagnosis and delayed diagnosis is common and contributes to patient distress and distrust, while simultaneously limiting opportunities for prevention and intervention. 8 Even though some data show more depression and anxiety among women who are aware of their condition, 4 it seems as if the PCOS diagnosis itself is not a cause of distress for women with PCOS, but rather the related comorbidities. 9 Overdiagnosis or misdiagnosis, on the other hand, may generate anxiety regarding potential risks for infertility, diabetes, cardiovascular disease, and obesity. 8

## Polycystic Ovary Syndrome Diagnosis

**Fig. 1:**
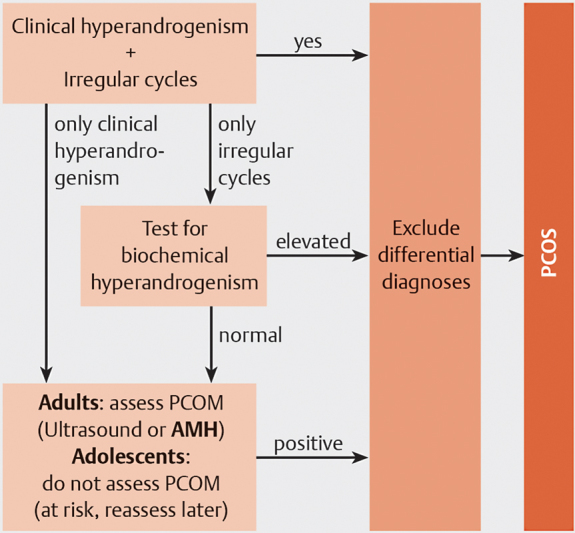
Diagnostic workup of polycystic ovary syndrome (PCOS) according to 2023 international evidence-based guideline for the assessment and management of polycystic ovary syndrome, including anti-Müllerian hormone (AMH) as an alternative diagnostic method for polycystic ovary (PCO). PCOM, polycystic ovary morphology.

The 2023 updated international evidence-based guideline for the assessment and management of PCOS recommends using the Rotterdam criteria for the diagnosis of PCOS, but in this updated guideline these criteria are now better specified and evidence-based. 1 For diagnosis in adults, two out of the following three criteria are required: (1) ovulatory dysfunction (OD); (2) clinical or biochemical hyperandrogenism (HA); and (3) polycystic ovary morphology (PCOM). In addition, other causes, for example, hypothyroidism and hyperprolactinemia, should be excluded. In adolescents, both OD and HA are required, whereas the PCOM criterion should not be used due to low specificity in this age group. The flow chart for diagnosis is shown in Fig. 1 .

### Ovarian Dysfunction

The OD criterion indicates oligo- or anovulation, with irregular menstrual cycles as the most common symptom. In the updated 2023 PCOS guideline, criteria for OD are defined as a menstrual cycle < 21 or > 35 days, 3 years after menarche until perimenopause. Adolescents within 3 years after menarche need special consideration, as reviewed in the guideline. 1

### Hyperandrogenism

HA can be assessed either clinically or biochemically. Regarding clinical HA, hirsutism is highly correlated with biochemical HA, whereas acne and alopecia are less specific. For objective assessment of hirsutism, the use of the modified Ferriman–Gallwey score is recommended, recognizing that self-treatment is frequently employed. 10 In these cases, self-reported scores can be used. If clinical HA is not present, biochemical HA can be determined. Biochemical HA is best assessed using total testosterone or free androgen index analyzed with highly accurate tandem mass spectrometry (liquid chromatography with tandem mass spectrometry) assays.

### Polycystic Ovary Morphology

The assessment of PCOM has until recently been done by ultrasonography but in the 2023 international PCOS guideline, measurement of serum anti-Müllerian hormone (AMH) has been added as an alternative. Neither method is recommended to be used within 8 years after menarche, due to low specificity.

Ultrasound is at present still the primary method for assessing PCOM in most clinical settings; however, it is expensive, and the availability is often limited or even absent. According to the diagnostic criteria as defined in the evidence-based guideline, the cut-off for PCOM is ≥ 20 follicles with a diameter of 2 to 9 mm in at least one ovary on transvaginal ultrasonography. 1 As alternatives, the ovarian volume or follicle number per cross-section can be used, with a cut-off of ≥10 mL for ovarian volume and ≥10 follicles per cross-section. If abdominal ultrasound is used, ovarian volume should be the assessment method of choice.

As AMH strongly correlates with antral follicle count (AFC) on ultrasound, the 2023 guideline has now incorporated this as an alternative method to estimate PCOM. 1

### Phenotypes, Other Diagnostic Criteria

The Rotterdam criteria/ international evidence-based diagnostic criteria results in four different possible phenotypes in adults: A: HA + OD + PCOM; B: HA + OD; C: HA + PCOM; and D: OD + PCOM, each encompassing different hormonal and metabolic profiles. 11 The differences in consequences depending on phenotypes are not yet fully understood, especially regarding long-term outcomes. According to a Finnish multicenter study, AMH levels were highest in PCOS patients with phenotype A. 12

Over time, other diagnostic criteria have been used; two of these are still applied to some extent. The National Institutes of Health (NIH) criteria require HA and oligo-amenorrhea and corresponds to phenotypes A and B but does not include PCOM. 13 The Androgen Excess PCOS Society (AE-PCOS) diagnostic criteria, on the other hand, involve essential androgen excess and, additionally, either oligo/amenorrhea or PCOM, thus corresponding to phenotypes A, B, and C but not D. 14

Since the diagnostic criteria differ and include different phenotypes, clinical presentation differs and contributes to significant heterogeneity. This is further influenced by variation across the life course, symptoms influenced by excess weight, and ethnic diversity. As a result, both diagnosis and treatment of PCOS are challenging, and can lead to delayed diagnoses, poor diagnostic experiences, and dissatisfaction with care. 11

## AMH as Marker of Ovarian Reserve

AMH is a glycoprotein that belongs to the transforming growth factor-β family, and in women it is secreted from the granulosa cells of preantral and small antral follicles in the ovary. AMH is absent in primordial as well as larger (>8 mm) antral follicles. 15 In the ovary, AMH acts as a gatekeeper, inhibiting the recruitment of primordial follicles from the follicle pool also regulating ovarian folliculogenesis by inhibiting FSH action on the follicles. 16 Recently, some animal and human data have suggested AMH having a role in GnRH-neuron signaling. 17 18

AMH has been shown to correlate well with the ovarian preantral and small antral follicles and can thus serve as a surrogate measurement of ovarian reserve as well as for the AFC to assess PCOM. 16 AMH levels are two- to threefold higher in women with PCOS. 19 AMH levels have also been used to predict menopause, as menopause occurs when the ovarian pool reaches a critically low level. Together with age, AMH levels can be adjusted for the prediction of menopausal age, and it has been shown that women with low age-specific AMH levels reach menopause earlier than those with high age-specific levels. 20 21

Assessment of AMH is currently used as part of the evaluation before in vitro fertilization (IVF) and can predict ovarian response to gonadotrophin stimulation. While AMH is a predictor of oocyte yield after controlled ovarian hyperstimulation in an IVF treatment, no convincing evidence exist of AMH being a valid predictor of oocyte quality or the chance of achieving natural pregnancy. 22

## Factors Affecting AMH Levels

The 2023 updated PCOS guideline has included measurement of AMH as an alternative to ultrasound when assessing PCOM. This enables general practitioners, endocrinologists, and other health personnel who do not have access to ultrasound, to assess PCOM and will hopefully lead to more effective and continuous overall care, while also saving resources for specialized care if the diagnostic workup could be mainly concentrated on primary care. Given the complexity of PCOS, the guideline does not support using serum AMH as a sole marker for PCOS. 23

Healthcare professionals also need to be aware that there are several factors that might influence AMH in the general population, including laboratory assays, age, body mass index (BMI), ethnicity, menstrual cycle stage, pregnancy, use of oral contraceptive pill, and ovarian surgery. 2 24 The effects of these factors on the AMH level are described in Table 1 .

**Table 1:** Factors influencing AMH levels

	Change in AMH
Increasing age	Lower
Increasing body mass index	Lower
Ethnicity	
Caucasian	Higher
Asian, Hispanic, Afro-Americans	Lower
Menstrual cycle stage	Indifferent/Clinically nonsignificant
Pregnancy	Lower
Use of hormonal contraceptive	Lower
Ovarian surgery	Lower

Abbreviation: AMH, anti-Müllerian hormone.

### Assay

The updated PCOS guideline recommends that laboratories involved in AMH measurements in females should use population and assay-specific cut-offs. 1 Commercial assays for the measurements of AMH have been available since the late 1990s, the AMH Gen II ELISA (marketed by Beckman-Coulter, Inc.) being the most widely used assay kit for many years. In recent years, however, new AMH measurement kits have become available, including the Elecsys AMH Immunoassay (Roche Diagnostics International Ltd, Indiana), Ultra-Sensitive AMH/MIS ELISA kit (Ansh Labs, Texas), and the automated Access AMH assay (Beckman-Coulter Diagnostics, California).

A study by Li et al evaluated the three newer AMH assay methods, namely, the Access AMH assay, Elecsys AMH Immunoassay, and Ultra-Sensitive AMH/MIS ELISA, and compared them to the older AMH Gen II ELISA. Results showed that values obtained from the Elecsys AMH Immunoassay were lower than the Gen II and Access AMH assays (0.88-fold and 0.86-fold, respectively). 25 These findings were also confirmed by Moolhuijsen et al. 26 AMH values obtained with the Ultra-Sensitive AMH/MIS ELISA were higher than those obtained with the Gen II and Access AMH assays (1.77-fold and 1.65-fold, respectively). 25 It is important to acknowledge that clinical cut-off for AMH in PCOS would acquire assay-specific cut-offs. 27

### Age

The concentration of serum AMH is dependent on the number of remaining oocytes in the ovaries. 2 Follicle development varies across the lifespan and is increased in adolescence, after which the number falls subsequently until menopause, when oocytes are depleted. According to previous studies, age-based decline in AMH is also known to be much less pronounced in PCOS women compared with controls. 28 29 There is thus a need for age-specific cut-offs for both PCOM and AMH. According to a systematic review, the sensitivity and specificity suggests greater accuracy of AMH in PCOS diagnosis in adults than in adolescents. Thus, the updated PCOS guideline recommends that PCOM should not be used in adolescent or young adults 8 years or less from menarche. 1

### Body Mass Index

Obesity is more common in women with PCOS compared with women without PCOS and a recent study showed that women with PCOS gain more weight annually compared with women without PCOS. 30 Previous studies have found a strong correlation between decreasing AMH levels and increasing BMIs in patients both with and without PCOS, suggesting different AMH cut-offs for different BMI groups. 31 32 Indeed, a recent study looking at different BMI subgroups and correlation between oligo-anovulation and AMH suggested progressively lower AMH cut-offs for women with increasing BMI to diagnose PCOS. 33

### Ethnicity

Previous studies have suggested a variation in AMH according to ethnicity. One study comparing Chinese and European women from the Netherlands, Belgium, Germany, France, and Turkey found that from the age of 25 years onward, Chinese women had significantly lower AMH than women of European origin. 34 This was confirmed in another study, where Chinese women had a lower AMH cut-off value for diagnosing PCOS compared with non-Asian women. 35 When comparing Caucasian women to Afro-Americans and Hispanic women, AMH levels were highest in Caucasian women. The clinical impact of these differences may be substantial and one of the studies found the natural menopause being 1 to 2 years earlier in Chinese women compared with European. 34

### Menstrual Cycle Stage

Some fluctuations of serum AMH levels according to menstrual cycle stage have been demonstrated in previous studies, but generally these changes are considered clinically irrelevant for the estimation of the ovarian reserve in individual woman. 36 One recent study measured AMH levels in 47 women every second day during two menstrual cycles. 37 All participants had a regular menstrual cycle, a BMI between 19 and 26 kg/m ^2^ , and were 18 to 40 years old. The study showed that inter-participant and intra-cycle variability of serum AMH levels were larger than inter-cycle variability and hence were in line with previous findings. It is, however, important to remember that these studies were performed in the general population, not specifically on women with PCOS. 37

### Pregnancy

Women with PCOS have higher AMH levels also during pregnancy compared with non-PCOS women. 38 39 40 41 There have been conflicting results on AMH kinetics in pregnancy, with some studies finding that AMH remained stable, 42 whereas others found a decrease in AMH as the pregnancy progressed. 43 A recent systematic review, consisting of eight studies and 1,719 participants, found an association between reduced maternal AMH and advancing gestational age. 44 These results were confirmed in a prospective, longitudinal cohort study where the median difference of AMH was –39.8% between the first and second trimester of pregnancy. 45 However, in postpartum, increased AMH levels were found when comparing with AMH levels during pregnancy. These findings should be taken into consideration when assessing PCOS in pregnant women, especially during last two terms.

### Use of Hormonal Contraceptive

Results on the effect of hormonal contraceptive use on AMH levels have been conflicting, with some studies finding up to 55% reduction of AMH levels in contraceptive users compared with controls, whereas other studies have found no difference in AMH between the two groups. 46 A large American study, comprising of 27,125 participants, aged 20 to 46 years, found that women using oral contraceptive pills, implants, or vaginal ring had the largest reduction in AMH levels compared with those not using contraceptives (−24%, −23%, and −22%, respectively). In a Finnish prospective spin-off study, it was also observed that continuous use of all combined contraceptives decreased AMH levels significantly during 9 weeks of treatment and decease was of same magnitude for contraceptive patch, pill, and vaginal ring. 47

Women using progesterone only pills or hormonal intrauterine devices had smaller reductions in AMH compared with those not using contraceptives (−15% and −7%, respectively). Among those using oral contraceptive pills, duration of contraceptive use (ranging from 1 month to 20 years) was not associated with a further decrease of AMH levels. 46

### Ovarian Surgery

In fertile aged women, a conservative ovarian cyst enucleation, with as little damage as possible on the normal ovarian tissue, is the preferred surgical intervention for the treatment of benign ovarian cysts. Even though cyst enucleation techniques aim at being fertility-sparing, conservative surgery will affect to some extent AMH and fertility, due to unavoidable removal of normal ovarian tissue or surgical damage to the remaining normal ovarian tissue. 48 A recent Swedish study on 75 fertile-aged women showed that type of cyst might also play a role. In this study, AMH decreased more in women with endometriomas than in women with dermoid cysts. 49

## Utility of AMH in PCOS Diagnosis

The utility of AMH in PCOS diagnosis has been an area of interest for a long time. Some of the relevant studies are summarized in Table 2 . 35 50 51 52 53 54 55 56 57 58 59 60 61 62 63 Given that serum AMH levels have been shown to be two to three times higher in women with PCOS, it has been proposed as an alternative tool for the diagnosis of PCOS. 19 28 60 64 However, use of AMH as a single marker for PCOS has poor sensitivity and specificity 58 61 62 and therefore AMH as a single marker for PCOS is not recommended. 1

**Table 2:** Studies assessing AMH cut-off values for PCOM and/or PCOS

Author	Outcome	Main results	Study population (including PCOS criteria)	PCOM criteria	Study setting	AMH cut-off value	AMH assay used
Sumji et al 63	PCOS	In a ROC analysis, the cut-off for AMH of 3.76 ng/mL had sensitivity of 86.7% and specificity of 62.7%	Women presenting symptoms suggestive of PCOS, aged 18–35. *N* (total) = 188, *N* (PCOS) = 113. Rotterdam criteria	≥ 10 peripheral follicles measuring 2–8 mm and/or ovarian volume > 10 mL	Case–control study. India	3.76 ng/mL	ELISA (Ansh Labs, Texas), manual
Piltonen et al 59	PCOM PCOS	AMH tested as a surrogate for PCOM. Cut-offs: 95%, 97.5%, 5 and 3.2 ng/mL. AMH cut-offs resulted in 5.9, 6,8, 9.8, and 13.6% prevalence of PCOS, respectively. All cut-offs captured populations with typical characteristics for PCOS as for hormonal and metabolic outcomes. AMH cut-off for PCOS (NIH criteria) was 4.9 ng/mL	Population-based, all aged 31 y. *N* (total) = 2,917, *N* (PCOS) = 171–395. Rotterdam criteria	No ultrasound data available	Population-based birth cohort study. Cross-sectional. Finland	10.35 ng/mL (97.5 percentile), 8.10 ng/mL (95 percentile), 5 ng/ml, 3.2 ng/mL	ECL, Elecsys AMH assay (Roche Diagnostics, Germany), automated
Zhang et al 35	PCOM PCOS	In both PCOS and PCOM, obese individuals showed the lowest AMH levels, whereas underweight ones had the highest.	Infertile patients aged 21–35 years. PCOS were diagnosed by modified Rotterdam criteria (OA + HA or OA + PCOM). N(total) = 15 970, N(PCOS) = 3775, N(PCOM) = 2879.	TVS: ≥12 follicles measuring 2–9 mm in diameter. PCOM group included PCOM with no HA or OA.	Cross sectional study. China.	4.45 ng/mL for total population. BMI <18kg/m2: 5.145 ng/mL; BMI 18 -24kg/m2: 4.345 ng/mL; BMI 25- < 28 kg/m2: 4.115 ng/mL; BMI ≥ 28kg/m2: 3.165 ng/mL	ECL, Elecsys AMH assay (Roche Diagnostics, Germany), automated
Bell et al 52	PCOM	AMH ≥ 44.0 pmol, suggested by the ROC curve, identified 80.6% of women with PCOM, falsely identified 15.2%. AMH BA2 assay cut-off of ≥ 33.2 pmol/L offered 80.6% sensitivity and 79.5% specificity for PCOM	163 non–healthcare-seeking women aged 18–39 y. Rotterdam criteria	≥ 25 follicles in at least one ovary	Cross-sectional study. Two different assays were used, cut-offs that most accurately identified women with PCOM were determined using ROC curves. Australia	≥ 33.2 pmol/l	Immunoassay, Beckman Access 2 assay (Beckman Coulter, Australia), pico Ansh assay (Ansh Labs, Texas), manual
Dietz de Loos et al 54	PCOM	For PCOM, an AMH cut-off of 3.2 ng/mL had sensitivity 88.6%, specificity 84.6%. PCOS phenotype A had ROC AUC of 93.6%	Median age PCOS 29.0 y, controls 36.0 y. *N* (total) = 2014; *N* (PCOS development) = 484, *N* (controls development) = 575, *N* (PCOS validation) = 455, *N* (controls, validation) = 500. Rotterdam criteria	AFC ≥ 12/ovary or/and women with an ovarian volume of > 10 mL	Case–control study. AMH cut-off was established and validated in separate cohorts. The Netherlands	3.2 ng/mL	ECL, Elecsys AMH Plus and Elecsys AMH assay (Roche Diagnostics, Germany), automated
Ramezani Tehrani et al 60	PCOS	The thresholds for predicting PCOS within the age groups of 20–27, 27–35, and 35–40 y were 5.7, 4.55, and 3.72 ng/mL, respectively	PCOS group recruited from a reproductive endocrinology research center, controls selected from a cohort study. Age 20–40 y. *N* (total) = 803, *N* (PCOS) = 303, and *N* (eumenorrheic non-hirsute control) = 500. Rotterdam criteria	≥12 follicles measuring 2–9 mm in diameter in each ovary and/or ovarian volume of > 10 mL	Cross-s sectional study. Iran	5.7, 4.55, and 3.72 ng/mL	Immunoassay, Gen II Kit (Beckman Coulter, California), manual
Bansal et al 51	PCOM PCOS	AMH cut-off at 5.1 ng/mL (sensitivity 70.97% and specificity 82.02%), predicted PCOS and correlated with PCOM	Women with acne recruited from a dermatology unit of a tertiary care hospital, aged ≥25 y. *N* (total) = 120, *N* (PCOS) = 31. Rotterdam criteria	AFC ≥ 12/ovary or/and women with an ovarian volume of > 10 mL	Prospective study. Cut-off value for AMH was calculated, determined by the ROC curve. India	> 5.1 ng/mL	Immunoassay system (DXI-600, Beckman Coulter, California), (DKO004, Diametra, Italy), automated
Ahmed et al 50	PCOM PCOS	Determined by the ROC curve, AMH > 3.19 ng/mL was substantially correlated with PCOM with a sensitivity of 72% and specificity of 70%	Patients from the obstetrics and gynecology clinics aged 18–38 y. *N* (total) = 148, *N* (PCOS) = 79. Rotterdam criteria	AFC ≥ 12 measuring 2–9 mm in diameter in one ovary	Case–control study assessing the occurrence of PCOS using an AMH suggested by the ROC curves. Saudi Arabia	3.19 ng/mL (determined by ROC curve)	ELISA (Ansh Labs, Texas), manual
Saxena et al 62	PCOM PCOS	The cut-off for maximum diagnostic potential of AMH alone for PCOS was 3.44 ng/mL, with sensitivity of 77.78% and specificity of 68.89%. Median AMH level was 4.32 ng/mL in PCOS cases and 2.32 ng/mL in controls	Women aged 18–35 y attending the Gynecology OPD of Dr. RML Hospital, New Delhi. *N* (total) = 90, N (PCOS) = 45. Rotterdam criteria	AFC ≥ 12 measuring 2–9 mm in diameter in one ovary, or ovarian volume > 10 mL	Case–control study. PCOS cases and control were matched for age and BMI. India	3.44 ng/mL	ELISA (Immunoconcept Bio-Detect), manual
Matsuzaki et al 58	PCOS	Cut-off value for diagnosing PCOS was 7.33 ng/mL, identified through ROC curve analysis (sensitivity 44.7%, specificity 76.8%). A cut-off of 10 ng/mL exhibited high specificity (92.6%) but low sensitivity	Women with PCOS aged 18–48 y, and women with normal cycles (control group) aged 20–46 y. *N* (total) = 209, *N* (PCOS) = 114. Rotterdam criteria	AFC ≥ 12 measuring 2–9 mm in diameter in one ovary, or ovarian volume > 10 mL	Case–control study. Japan	7.33 ng/mL, 10 ng/mL	ECL, Elecsys AMH assay (Roche Diagnostics, Germany), automated
Lauritsen et al 56	PCOM PCOS	The prevalence of PCOS was 16.6% based on the Rotterdam criteria. When substituting the criterion for polycystic ovaries with AFC > 19 or AMH > 35 pmol/L, the prevalence of PCOS was 6.3 and 8.5%	Female healthcare workers aged 20–40 y. *N* (total) = 447. Rotterdam criteria	AFC ≥ 12 measuring 2–10 mm in diameter in one ovary, or ovarian volume > 10 mL	Cross-sectional cohort study between 2008 and 2010. Denmark	> 35 pmol/L	AMH/MIS kit (Beckman Coulter, France), manual
Sahmay et al 61	PCOS	Sensitivity and specificity for PCOS diagnosis with the combination of OA and/or HA with AMH were 83 and 100% according to the Rotterdam criteria; 83 and 89% according to the NIH criteria; and 82 and 93.5% according to the AES criteria	Women admitted to the gynecologic endocrinology department due to menstrual irregularities or symptoms of HA. *N* (total) = 606	AFC ≥ 12 measuring 2–9 mm in diameter in each ovary, or ovarian volume > 10 mL	Cross-sectional study. Turkey	3.8 ng/mL	AMH Gen II ELISA (Beckman Coulter, California), manual
Li et al 57	PCOS	The cut-off value for predicting PCOS was 3.92 ng/mL (sensitivity 65%, specificity 62%). For PCOS patients HA + , the cut-off was 4.23 ng/mL (sensitivity 82%, specificity 64%). For PCOS HA − , the cut-off was 3.76 ng/mL, (sensitivity 64%, specificity of 62%	PCOS women with oligo/amenorrhea, and ≥ follicles 2–9 mm in diameter per ovary. *N* (total) = 192, *N* (PCOS HA + ) = 62, *N* (PCOS HA − ) = 69. Rotterdam criteria	No ultrasound data available	Case–control study. ROC curves were generated to assess the diagnostic accuracy of AMH. China	3.92 ng/mL for all PCOS patients, 4.23 ng/mL for HA + , and 3.76 ng/mL for HA−	ELISA (Diagnostic Systems Laboratories, Texas), manual
Eilertsen et al 55	PCOM PCOS	When replacing PCOM with AMH, the specificity and sensitivity for identifying PCOS were 97.1 and 94.6%, respectively, according to the Rotterdam criteria and 97.2 and 95.5% according to the AES criteria	Women with prior preterm birth and their controls from an earlier study. *N* (total) = 262, *N* (PCOS-Rotterdam criteria) = 56, *N* (PCOS-AES) = 44	AFC ≥ 12 measuring 2–9 mm in diameter and/or ovarian volume ≥10 mL in at least one ovary	Data from a cross-sectional, case–control. Norway	20 pmol/L	ELISA (Diagnostic Systems Laboratories, Texas), manual
Dewailly et al 53	PCOM PCOS	Determined by the ROC curve, areas under the curve for follicle number and serum AMH were 0.949 and 0.973 (sensitivity 81 and 92%, specificity 92 and 97%) using threshold values of 19 follicles and 5 ng/mL	Women with HA, menstrual disorders and/or infertility. *N* (total) = 240, *N* (non-PCOS with HA− and ovulatory cycles) = 105, *N* (PCOS with HA+ or oligo/amenorrhea) = 73, *N* (PCOS with HA+ and oligo/amenorrhea) = 62	No ultrasound data available	Case–control study. France	5 ng/mL	AMH-EIA (Beckman Coulter, France), manual

Abbreviations: AMH, anti-Müllerian hormone; ECL, electrochemiluminescence; ELISA, enzyme-linked immunosorbent assay; PCOM, polycystic ovary morphology; PCOS, polycystic ovary syndrome.

Studies conducted over the past two decades have indicated that serum AMH provides a practical and cost-effective biomarker for detecting PCOM, with AMH levels correlating with the number of antral follicles on ultrasound. In the first international PCOS guideline in 2018, evidence was not enough to recommend AMH as a diagnostic marker for PCOM. 23 65 In the recent 2023 guideline, 1 evidence had emerged and AMH is now an alternative tool to assess PCOM and is thus part of the diagnostic criteria ( Fig. 1 ).

As evident in Table 2 , there is currently not one single AMH cut-off to assess PCOM. As laboratory methods vary in different parts of the world, the updated PCOS guideline has recommended the use of population and assay-specific cut-offs. However, the traditional way of defining the “normal” range as a cut-off of within 2 standard deviations is not appropriate for defining diagnostic cut-offs for a clinical condition like PCOS. Here, more important considerations include clustering with other clinical features such as clinical HA, oligo-anovulation, or prediction of long-term health outcomes such as fertility. Unfortunately, large studies on PCOS with cluster analyses are lacking. A recent epidemiological study was, however, able to analyze different cut-offs for AMH and the relation to clinical outcomes showing that as low as 3.2 ng/mL suggested by Dietz de Loos et al 54 was able to detect women with PCOS with typical hormonal and metabolic profile. 59

As evident in Table 2 , different cut-offs have indeed been used to identify PCOM, emphasizing that the cut-off is dependent on the population and the assay used. Consequently, the sensitivity and specificity for PCOM also differ depending on the cut-off used, 50 52 53 54 and the cut-off will also affect the prevalence of PCOS. 55 56 59 Thus, in future studies, it is important to define how the AMH cut-off has been established. 23 59

Defining in which population AMH is assessed is also important; women with obesity have lower AMH levels also in PCOS 35 ; and younger women might need higher cut-offs than older women to define PCOM. 60

To summarize, AMH assessment is now an evidence-based alternative to assess PCOM in the diagnosis of PCOS. However, it is relevant only when using the Rotterdam criteria, which is also now evidence-based, or when using AE PCOS criteria, but not when using the NIH criteria where PCOM is not part of the diagnostic criteria.

## Conclusion

AMH serves as a new tool to diagnose PCOM. It will enable PCOS diagnosis in primary care and could facilitate early diagnosis, prevention, and support. There are, however, some limitations in the usage that should be acknowledged. These include physiological aspects such as age, ethnicity, and obesity and iatrogenic causes such as hormonal medication and ovarian surgery. Age and platform-related reference ranges are warranted to optimize the usage of AMH as part of the PCOS diagnosis workup.
